# A two-herb formula inhibits hyperproliferation of rheumatoid arthritis fibroblast-like synoviocytes

**DOI:** 10.1038/s41598-021-83435-2

**Published:** 2021-02-16

**Authors:** Ying-Jie Chen, Yu-Xi Liu, Jia-Ying Wu, Chun-Yu Li, Min-Min Tang, Lu Bai, Xiu-Qiong Fu, Jun-Kui Li, Ji-Yao Chou, Cheng-Le Yin, Ya-Ping Wang, Jing-Xuan Bai, Ying Wu, Xiao-Qi Wang, Zhi-Ling Yu

**Affiliations:** 1 Research and Development Centre for Natural Health Products, HKBU Shenzhen Research Institute and Continuing Education, Shenzhen, China; 2 Centre for Cancer and Inflammation Research, School of Chinese Medicine, Hong Kong Baptist University, Kowloon Tong, Hong Kong China; 3 Consun Chinese Medicines Research Centre for Renal Diseases, School of Chinese Medicine, Hong Kong Baptist University, Kowloon Tong, Hong Kong China

**Keywords:** Immunology, Rheumatology

## Abstract

Fibroblast-like synoviocytes (FLS) play a pathogenic role in rheumatoid arthritis (RA). STAT3 signaling is activated in FLS of RA patients (RA-FLS), which in turn causes RA-FLS hyperproliferation. RL is a traditional remedy for treating inflammatory diseases in China. It comprises Rosae Multiflorae Fructus and Lonicerae Japonicae Flos. A standardized ethanolic extract of RL (RLE) has been shown to exert anti-arthritic effects in collagen-induced arthritis (CIA) rats. Some constituents of RLE were reported to inhibit JAK2/STAT3 signaling in rat FLS. Here, we determined whether RLE inhibits FLS hyperproliferation, and explored the involvement of STAT3 signaling in this inhibition. In joints of CIA rats, RLE increased apoptotic FLS. In IL-6/sIL-6R-stimulated RA-FLS, RLE reduced cell viability and evoked cell apoptosis. In synovial tissues of CIA rats, RLE lowered the protein level of phospho-STAT3. In IL-6/sIL-6R-stimulated RA-FLS, RLE inhibited activation/phosphorylation of STAT3 and JAK2, decreased the nuclear localization of STAT3, and downregulated protein levels of Bcl-2 and Mcl-1. Over-activation of STAT3 diminished RLE’s anti-proliferative effects in IL-6/sIL-6R-stimulated RA-FLS. In summary, RLE inhibits hyperproliferation of FLS in rat and cell models, and suppression of STAT3 signaling contributes to the underlying mechanisms. This study provides further pharmacological groundwork for developing RLE as a modern anti-arthritic drug.

## Introduction

Rheumatoid arthritis (RA), a chronic inflammatory autoimmune disease, affects around 1% of the world population and degrades quality of life of those who have it^1^. Fibroblast-like synoviocytes (FLS) play a pathogenic role in RA. FLS of RA patients (RA-FLS) are characterized by hyperproliferation and resistance to apoptosis, which results in synovial hyperplasia^2^. Hyperproliferation of RA-FLS leads to the destruction of cartilage and bone of RA patients^2^. Signal transducer and activator of transcription 3 (STAT3) signaling is activated in RA-FLS, which in turn promotes proliferation and inhibits apoptosis of RA-FLS^3^. Elevated interleukin-6 (IL-6) is commonly found in sera and the synovial fluid of RA patients^4^. Binding of IL-6 to its receptors activates Janus kinase 2 (JAK2), an upstream kinase of STAT3, which leads to the phosphorylation of STAT3^5^. Phosphorylated STAT3 forms homodimers and then translocate into the nucleus to transcriptionally upregulate survival genes such as Bcl-2 and Mcl-1^3^. Therefore, STAT3 signaling has been proposed as a target for treating RA^6^. Although current anti-arthritic drugs, e.g. certolizumab and tofacitinib, can effectively alleviate inflammation and joint deterioration in RA patients, they have unacceptable side effects, including serious infections and tuberculosis^7^. Multi-component herbal medicines have been recognized as alternatives for treating RA^8^.

An herbal formula RL, comprising Rosae Multiflorae Fructus (RMF, the dried fruit of *Rosa multiflora* Thunb.) and Lonicerae Japonicae Flos (LJF, the dried newly bloomed flower or flower bud of *Lonicera japonica* Thunb.), was written by a traditional Chinese medicine (TCM) doctor Sun Simiao in c. 652 A.D. (Tang dynasty of China) for managing inflammatory and/or infectious diseases^9,10^. RMF^11^ and LJF^12,13^, owing to their low toxicity, are consumed as nutraceuticals in diverse cultures. They are also used as folk remedies for treating RA^10,14–16^. Pharmacological studies showed that RMF^17^ and LJF-containing formulas^18,19^ can attenuate collagen-induced arthritis (CIA). We previously found that a standardized ethanolic extract of RL (RLE for short) has anti-RA effects in CIA rats^20^. Chlorogenic acid, a compound used for controlling RLE’s quality^20^, has been shown to be able to inhibit the proliferation of IL-6-stimulated rat FLS and suppress JAK2/STAT3 signaling^21^. In this study, we investigated the effects of RLE on apoptosis of FLS in joints of CIA rats, and on proliferation of, and apoptosis in, interleukin-6/soluble interleukin-6 receptor (IL-6/sIL-6R)-stimulated RA-FLS. We also investigated the involvement of STAT3 signaling in these effects.

## Results

### RLE increases apoptotic FLS in joints of CIA rats

**Figure 1:**
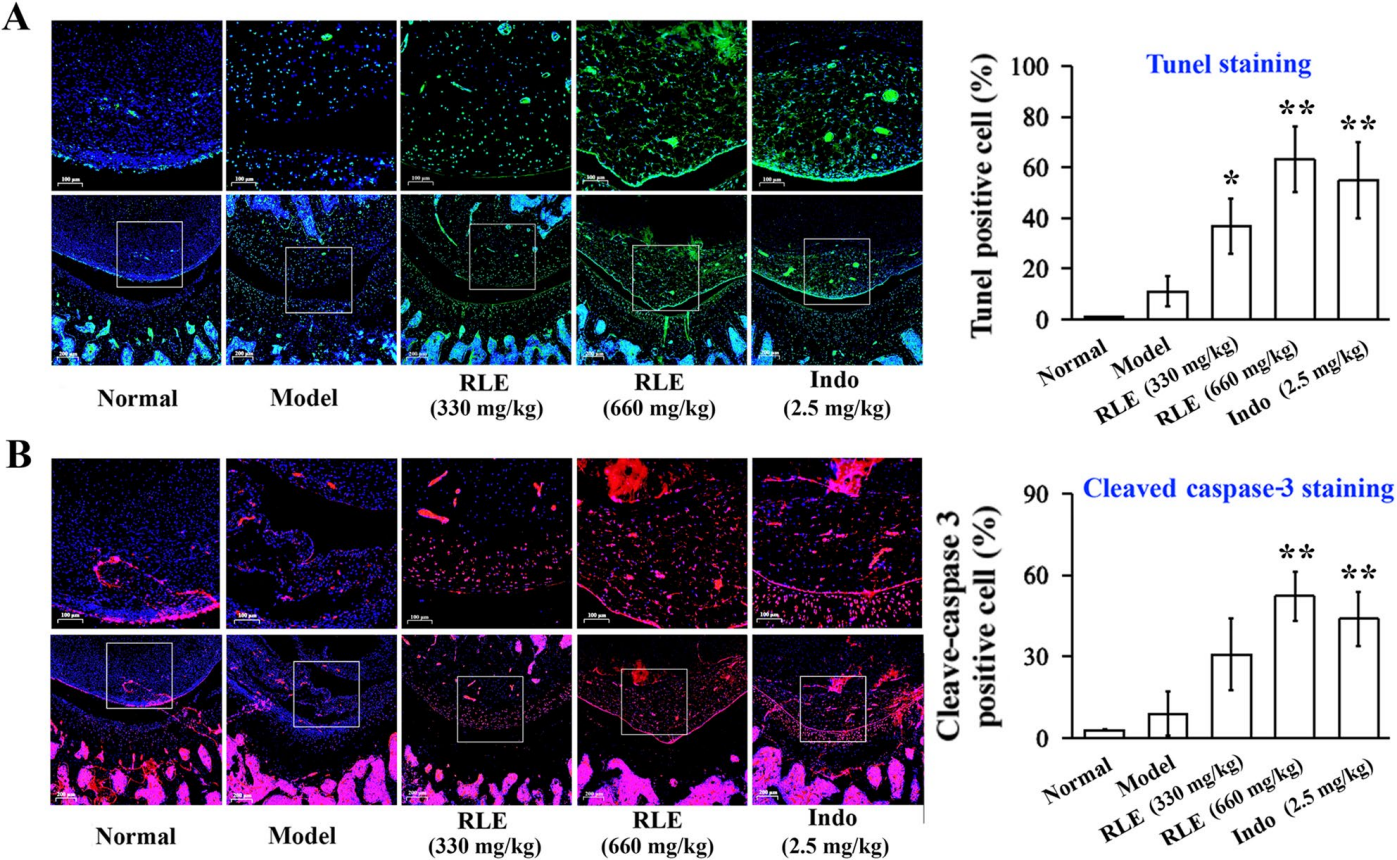
RLE increased apoptotic FLS in joints of CIA rats. (**A**) RLE increased TUNEL-positive FLS in joints of CIA rats. Representative images of TUNEL-stained joint sections are shown in left panels. Enlarged images (areas of synovium, left upper panels, scale bar: 100 μm) were magnified from the framed region in the corresponding large-scale images (left lower panels, scale bar: 200 μm). DAPI-positive (blue) cells and TUNEL‐positive (green) cells in enlarged images were counted using ImageJ software. Percentages of TUNEL-positive cells are presented in the right panel. (**B**) RLE increased cleaved caspase-3-positive FLS in joints of CIA rats. Representative images of cleaved caspase-3-stained joint sections are shown in left panels. Enlarged images (areas of synovium, left upper panels, scale bar: 100 μm) were magnified from the framed region in the corresponding large-scale images (left lower panels, scale bar: 200 μm). DAPI-positive (blue) cells and cleaved caspase-3‐positive (red) cells in enlarged images were counted using ImageJ software. Percentages of cleaved caspase-3-positive cells are presented in the right panel. In (**A**,**B**), rat joints were collected from a previous study (n = 6; randomly selected 6 from 8 rats in each group). *P < 0.05, **P < 0.01 *vs.* model group.

RA-FLS are resistant to apoptosis^2^. We examined the effect of RLE on apoptotic rate of FLS in joints of CIA rats using the TUNEL assay and immunohistochemistry (IHC) staining of cleaved caspase-3. DNA fragmentation resulting from apoptotic signaling cascades can be detected by TUNEL assays. Cleaved caspase-3 is an apoptosis marker. In normal and model groups, TUNEL-positive FLS (Fig. 1A) and cleaved caspase-3-positive FLS (Fig. 1B) in synovia of rat joints were very few, and there was no difference between the two groups. TUNEL assays showed that RLE dose-dependently and significantly induced apoptosis (Fig. 1A). In IHC staining assays, although RLE was not able to significantly increase apoptotic FLS at 330 mg/kg, it exerted significant effect at 660 mg/kg (Fig. 1B). The positive control indomethacin significantly increased TUNEL-positive FLS (Fig. 1A) and cleaved caspase-3-positive FLS (Fig. 1B) as well. These findings indicate that RLE promotes apoptosis of FLS in joints of CIA rats in a dose-dependent manner.

### RLE inhibits proliferation of, and induces apoptosis in, IL-6/sIL-6R-stimulated RA-FLS

**Figure 2:**
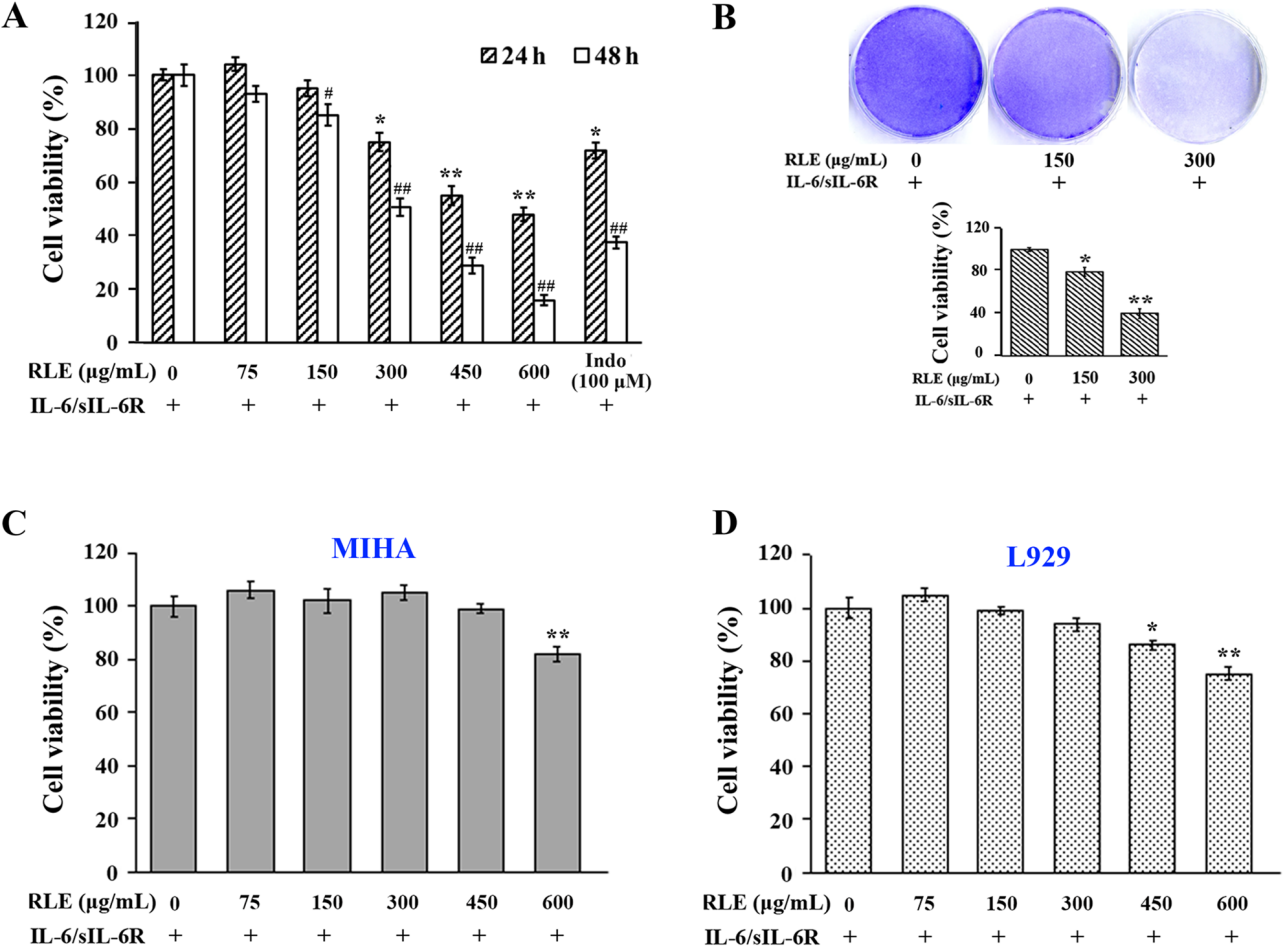
RLE reduced viability of RA-FLS. (**A**) CCK-8 assays in IL-6/sIL-6R-stimulated synoviocytes. Cell viability results are from 3 independent experiments. Viability of RLE-untreated cells was regarded as 100%. *P < 0.05, **P < 0.01 *vs.* RLE-untreated (24 h) group. #*P* < 0.05, ##*P* < 0.01 *vs.* RLE-untreated (48 h) group. (**B**) Crystal violet staining of IL-6/sIL-6R-stimulated synoviocytes. Representative images are shown in the upper panel, and quantitative results are shown in the lower panel (n = 3). (**C**) CCK8 assays in IL-6/sIL-6R-stimulated L929 cells. (**D**) CCK8 assays in IL-6/sIL-6R-stimulated MIHA cells. In (**C**,**D**), cell viability results are from 3 independent experiments. In (**B**–**D**), *P < 0.05, **P < 0.01 *vs.* RLE-untreated group whose cell viability was regarded as 100%.

**Figure 3:**
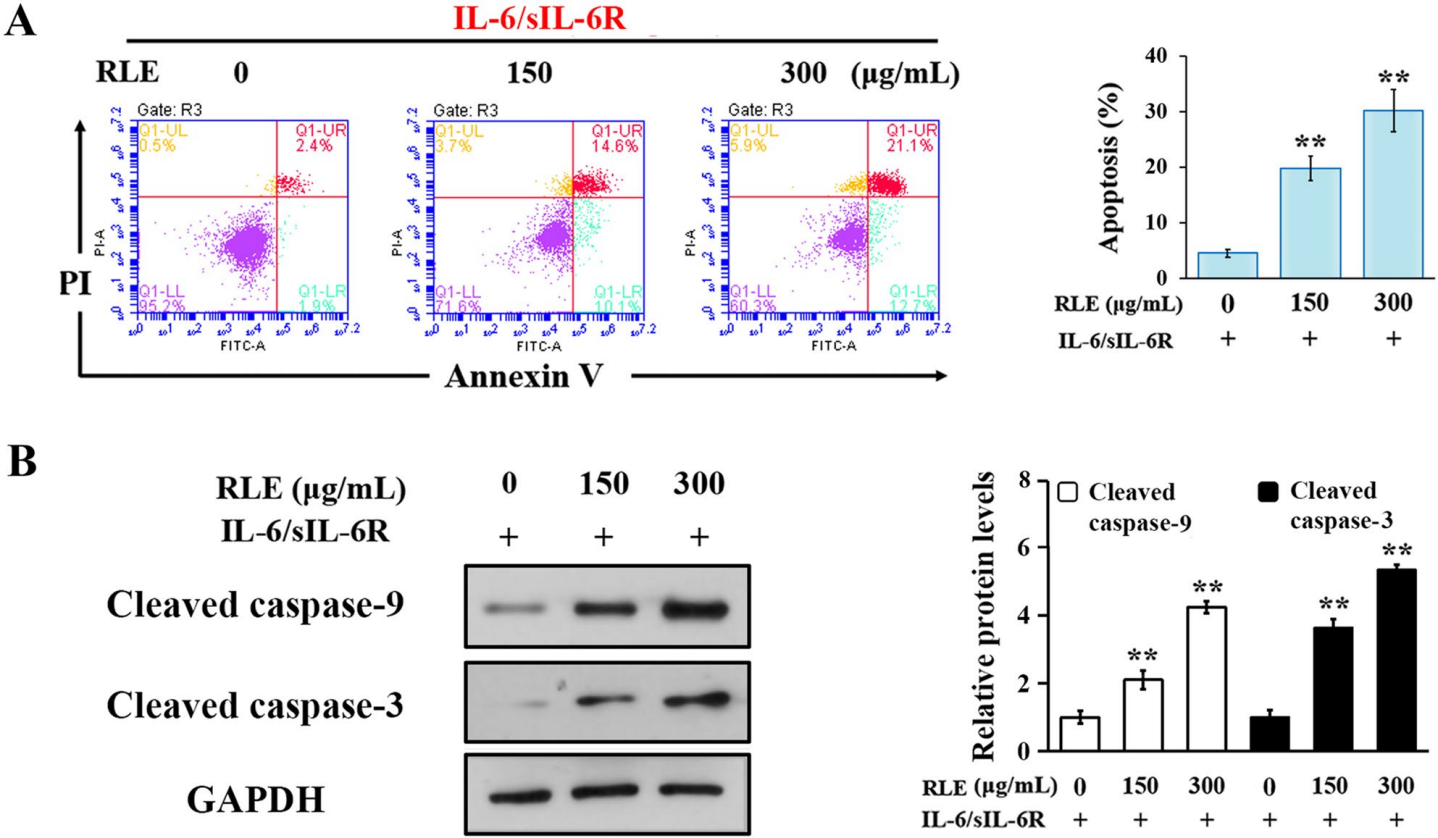
RLE induced apoptosis in RA-FLS. (**A**) RLE induced apoptosis in IL-6/sIL-6R-stimulated synoviocytes. Apoptosis was analyzed by flow cytometry after the cells were stained with Annexin V/PI. Representative scatter graphs are shown in left panels. FITC positive cells (right quadrants) were regarded as apoptotic cells. Quantitative results are shown in the right panel (n = 3). (**B**) RLE increased cleaved caspase-9 and cleaved caspase-3 in IL-6/sIL-6R-stimulated synoviocytes. Cells were pre-treated with indicated concentrations of RLE for 1 h and then treated with IL-6/sIL-6R for 24 h. Protein levels were examined using immunoblotting. Representative blots are shown in the left panel. Protein levels relative to GAPDH were analyzed using ImageJ software and are shown in the right panel (n = 3). In (**A**,**B**), *P < 0.05, **P < 0.01 vs. RLE-untreated group.

IL-6 signaling activates STAT3 in RA-FLS, which has been implicated in hyperproliferation and resistance to apoptosis of RA-FLS^3^. To determine the effects of RLE on proliferation of patient-derived synoviocytes, CCK-8 and crystal violet staining assays were performed. RLE dose- and time-dependently reduced viability of IL-6/sIL-6R-stimulated synoviocytes in CCK-8 assays (Fig. 2A). The anti-proliferative effects of 450 μg/mL of RLE were comparable to that of 100 μM of indomethacin (Fig. 2A). Crystal violet staining visualized RLE’s inhibitory effects on RA-FLS proliferation (Fig. 2B). To determine the effects of RLE on the proliferation of normal cells, mouse fibroblast L929 and human normal liver-derived MIHA cells were used. In the presence of IL-6/sIL-6R, the anti-proliferative effects of RLE in both L929 and MIHA lines were weak (Fig. 2C,D) and less potent than in RA-FLS (Fig. 2A,B). To determine the effects of RLE on apoptosis of synoviocytes, Annexin V-FITC/PI double staining assays were conducted. RLE dose-dependently induced apoptosis in IL-6/sIL-6R-stimulated synoviocytes (Fig. 3A). RLE’s apoptotic effects were verified by its ability to dose-dependently cleave caspase-3 and caspase-9 (Fig. 3B). As compared with the solvent treatment, IL-6/sIL-6R stimulation did not significantly affect synoviocyte apoptotsis (data not shown). These findings indicate that RLE inhibits proliferation of, and induces apoptosis in, IL-6/sIL-6R-stimulated RA-FLS.

### RLE lowers the protein level of p-STAT3 in synovia of CIA rats

**Figure 4:**
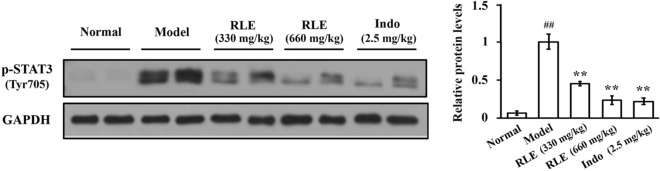
RLE lowered the protein level of p-STAT3 in synovial tissues of CIA rats. Rat tissues were collected in a previous study (n = 6; randomly selected 6 from 8 rats in each group). Protein levels were examined using immunoblotting. Representative blots are shown in left panels. Protein levels relative to loading controls were analyzed using ImageJ software, and are shown in right panels. ** *P* < 0.01 *vs.* model group. ## *P* < 0.01 *vs.* normal group.

In the mechanistic studies, we found that protein level of phospho-STAT3 (Tyr 705) in synovial tissues was higher in CIA rats than in normal rats, and RLE treatments significantly and dose-dependently down-regulated the level of this protein (Fig. 4). Indomethacin also inhibited phosphorylation of STAT3 in synovia of CIA rats (Fig. 4), which is in line with a previous report^22^. These data indicate that RLE inhibits STAT3 activation in synovia of CIA rats.

### RLE inhibits STAT3 signaling in IL-6/sIL-6R-stimulated RA-FLS

**Figure 5:**
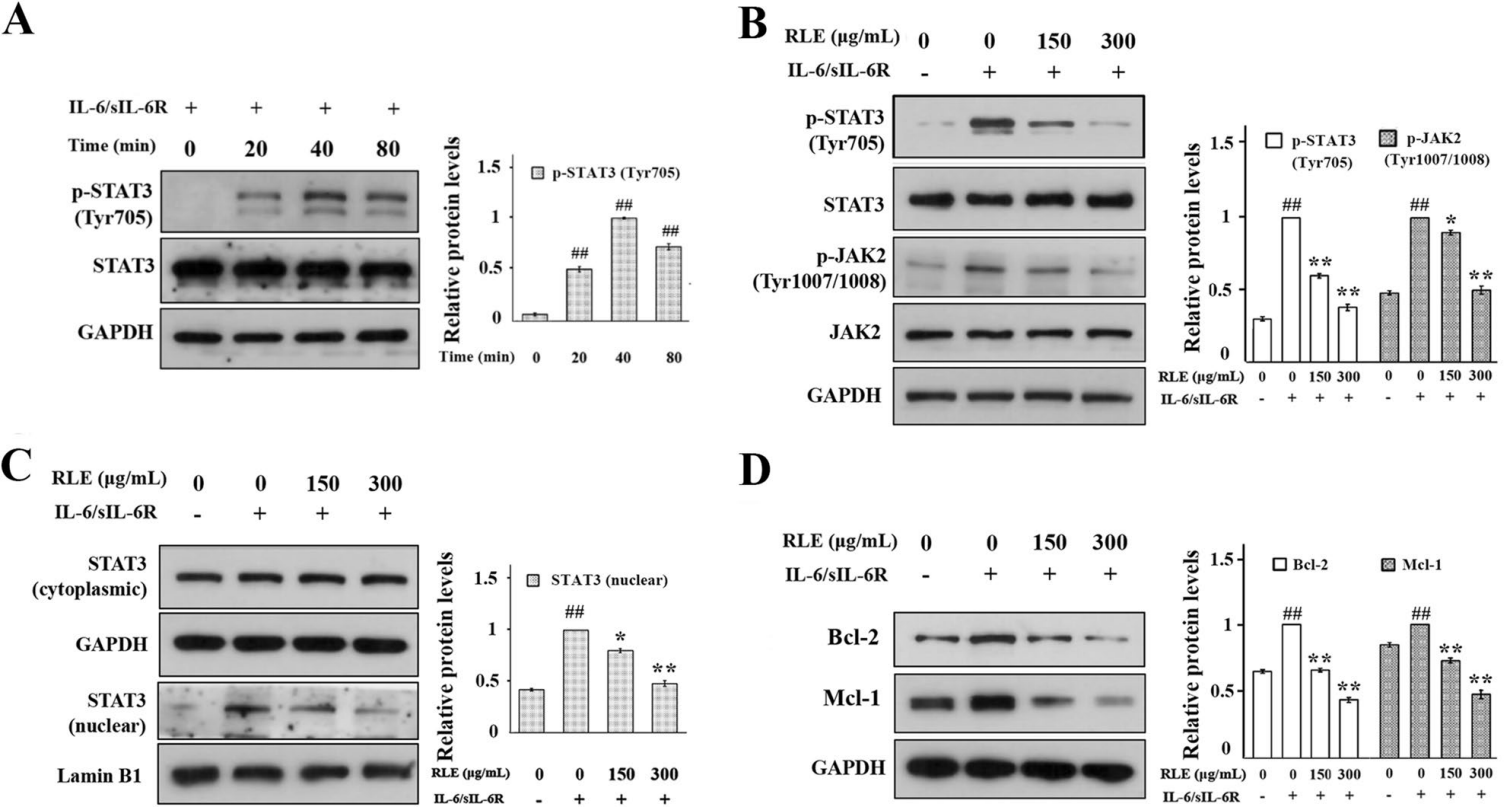
RLE inhibited STAT3 signaling in RA-FLS. (**A**) IL-6/sIL-6R time-dependently induced phosphorylation of STAT3 in RA-FLS. Synoviocytes were treated with IL-6/sIL-6R for indicated durations. (**B**) RLE suppressed the phosphorylation of STAT3 and JAK2 in IL-6/sIL-6R-stimulated synoviocytes. Cells were pre-treated with indicated concentrations of RLE for 1 h and then treated with or without IL-6/sIL-6R for 40 min. (**C**) RLE suppressed IL-6/sIL-6R-induced STAT3 nuclear localization in synoviocytes. Cells were treated as in (**B**). (**D**) RLE lowered protein levels of Bcl-2 and Mcl-1 in IL-6/sIL-6R-stimulated synoviocytes. Synoviocytes were pre-treated with indicated concentrations of RLE for 1 h and then treated with or without IL-6/sIL-6R for 24 h. In (**A**–**D**), protein levels were examined using immunoblotting. Representative blots are shown in left panels. Protein levels relative to loading controls were analyzed using ImageJ software, and are shown in right panels (n = 3). In (**A**,**B**,**D**), GAPDH served as the loading control. In (**B**), Lamin B1 and GAPDH served as loading controls of nuclear and cytoplasmic extracts, respectively. In (**A**), ## *P* < 0.01 *vs.* 0-min time point group. In (**B**–**D**), * P < 0.05, ** *P* < 0.01 vs. IL-6/IL-6R-only-treated group. #*P* < 0.05, ## *P* < 0.01 vs. vehicle-treated control group.

In cell assays, IL-6/sIL-6R stimulation time-dependently promoted the activation/phosphorylation at site Tyr705 of STAT3 in RA-FLS, with a peak at 40 min (Fig. 5A). Synoviocytes stimulated with IL-6/sIL-6R for 40 min were used as the cell model in subsequent assays. Further cellular assays showed that RLE dose-dependently suppressed IL-6/sIL-6R-induced phosphorylation of STAT3 and JAK2 without affecting protein levels of STAT3 and JAK2 in synoviocytes (Fig. 5B). It was also observed that RLE decreased STAT3 nuclear localization induced by IL-6/sIL-6R (Fig. 5C); and reversed IL-6/sIL-6R-induced up-regulation of protein levels of Mcl-1 and Bcl-2 (Fig. 5D) in synoviocytes. These results indicate that RLE inhibits STAT3 signaling in RA-FLS.

### Over-activation of STAT3 diminishes RLE’s inhibitory effect on proliferation of IL-6/sIL-6R-stimulated RA-FLS

**Figure 6:**
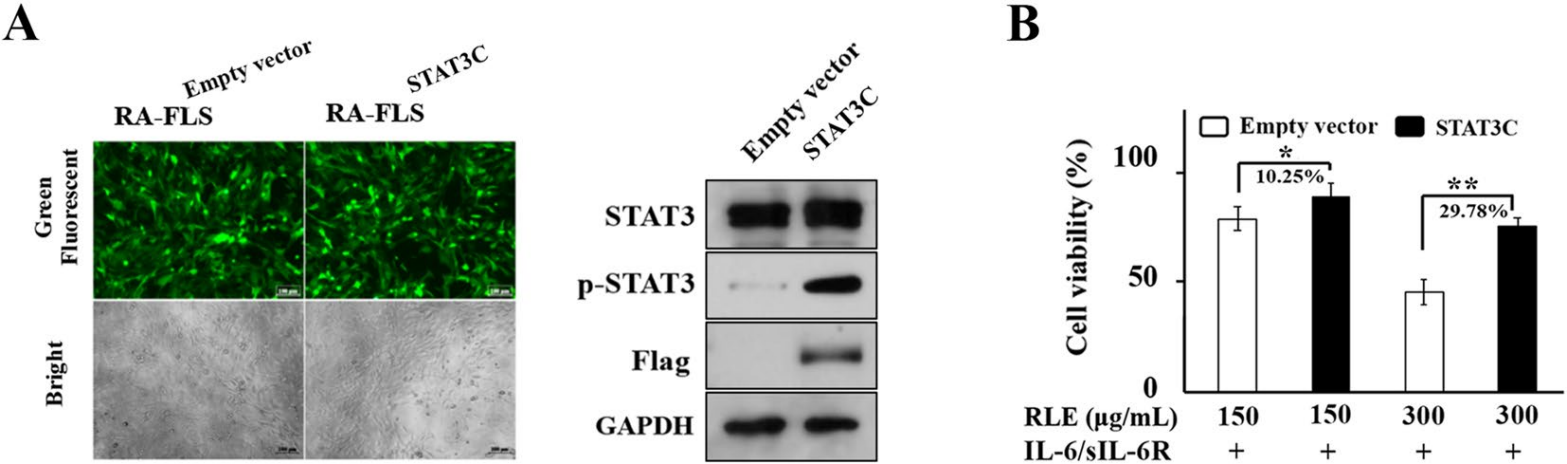
Over-activation of STAT3 diminished the anti-proliferative effect of RLE in RA-FLS. (**A**) Green fluorescent protein (GFP) expression, and protein levels of p-STAT3 (Tyr 705), STAT3 and Flag in RA-FLS^Empty vector^ and RA-FLS^STAT3C^. RA-FLS were transduced with empty vector or Flag-tagged STAT3C plasmid with GFP as a transduction indicator. Cells expressing GFP displayed green fluorescence. Representative green fluorescent and bright-field microscopy images of RA-FLS^Empty vector^ and RA-FLS^STAT3C^ are shown in the left panel. Scale bar: 100 μm. Protein levels of p-STAT3 (Tyr 705), STAT3 and Flag were determined using immunoblotting. Representative immunoblots are shown in the right panel. (**B**) Over-activation of STAT3 diminished the anti-proliferative effect of RLE in IL-6/sIL-6R-stimulated RA-FLS. Cells were transduced with Ad-Empty vector (RA-FLS^Empty vector^) or Ad-STAT3C (RA-FLS^STAT3C^). Cell viability was assessed using CCK-8 assays (n = 3). Differences of relative viabilities between RLE-treated RA-FLS^Empty vector^ and RLE-treated RA-FLS^STAT3C^ were calculated. * *P* < 0.05, **P < 0.01.

To determine whether STAT3 signaling plays a role in RLE’s effects on proliferation of RA-FLS, we over-activated STAT3 in RA-FLS by transducing STAT3C (A662C, N664C mutant), a constitutively active STAT3 variant^23^, into the cells. Microscopic analyses showed that synoviocytes were successfully transduced (Fig. 6A). Immunoblotting showed that transduction of STAT3C plasmid caused a remarkable elevation in levels of STAT3 and phospho-STAT3 (Tyr705) (Fig. 6A), showing an over-activation of STAT3. Upon STAT3 over-activation, inhibitory effects of 150 and 300 μg/mL of RLE on the proliferation of IL-6/sIL-6R-stimulated synoviocytes were decreased 10.25% and 29.78%, respectively (Fig. 6B). These findings indicate that inhibition of STAT3 signaling contributes to RLE’s inhibitory effect on RA-FLS proliferation.

## Discussion

In a previous study, we found that RLE reduces paw swelling and arthritis clinical scores, and alleviates bone destruction in CIA rats without observable adverse reactions^20^. Moreover, RLE improves body weight gain of CIA rats^20^. In the present study, we found that RLE promotes apoptosis of pathogenic FLS in CIA rats (Fig. 1A,B), and inhibits proliferation (Fig. 2A,B) of, and induces apoptosis (Fig. 3A) in, IL-6/sIL-6R-stimulated RA-FLS. These findings of our previous and the present studies indicate that RLE has potential to be developed as an effective and safe modern drug for treating RA.

**Figure 7:**
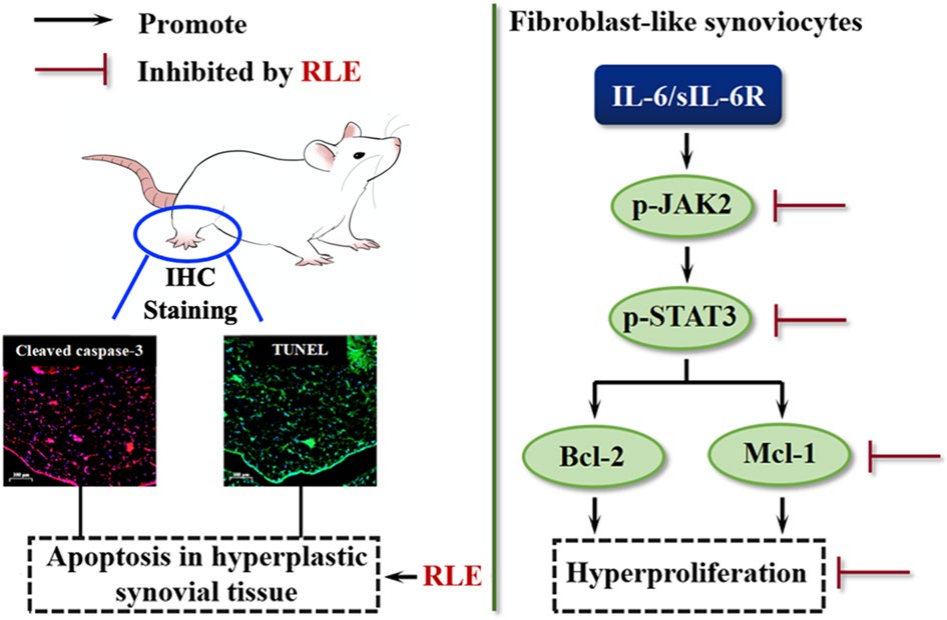
Effects of RLE on hyperproliferation of FLS.

We previously observed that RLE inhibits diverse toll-like receptor 4 (TLR4) signaling components in LPS-stimulated RAW264.7 and THP-1 cells^24^; and that RLE lowers the protein level of TLR4 and inhibits phosphorylation/activation of TLR4 downstream transcription factors, NF-κB p65 (nuclear factor-κB p65), AP-1 (activator protein-1) and IRF3 (interferon regulatory factor 3) in joints of CIA rats^20^. We also observed that RLE reduces production of pro-inflammatory cytokines IL-6, TNF-α and IL-1β which are regulated by transcription factors NF-κB, AP-1 and IRF3 in joint tissues and sera of CIA rats^20^. These findings indicate that inhibiting TLR4 signaling is one of the mechanisms of the anti-RA effects of RLE. Pro-inflammatory cytokines IL-6, TNF-α and IL-1β are also regulated by the transcription factor STAT3. These cytokines can be produced by RA-FLS^25^. It is reported that chlorogenic acid, a quality control marker of RLE^20^, can suppress JAK2/STAT3 signaling in rat FLS^21^. Thus, in this study, we investigated the involvement of STAT3 signaling in RLE’s inhibitory effects on FLS proliferation. We found that RLE inhibits STAT3 activation in synovia of CIA rats (Fig. 4) and suppresses STAT3 signaling in cell models (Fig. 5B–D). Over-activation of STAT3 attenuates the anti-proliferative effects of RLE in IL-6/sIL-6R-stimulated synoviocytes (Fig. 6B). Results of the present study indicate that inhibiting STAT3 signaling to suppress RA-FLS hyperproliferation is another anti-RA mechanism of RLE (Fig. 7).

RA-FLS play a key role in RA-associated bone erosion^26^. In RA-FLS, IL-6/sIL-6R induces expression of receptor activator of nuclear factor kappa-B ligand (RANKL), which is crucial for osteoclastogenesis^27^. In a previous study, we found that RLE alleviates bone erosion in CIA rats^20^. Further studies are warranted to determine whether RLE inhibits RANKL expression in IL-6/sIL-6R-stimulated RA-FLS by inhibiting the STAT3 pathway.

In summary, we for the first time found that RLE has pro-apoptotic effect in FLS of CIA rats, and has anti-proliferative and pro-apoptotic effects in IL-6/sIL-6R-stimulated RA-FLS; and that inhibition of the STAT3 signaling pathway contributes to the underlying mechanisms (Fig. 7). This study provides further pharmacological justifications for the traditional use of RL in treating inflammatory diseases, and provides additional pharmacological groundwork for developing RLE as a modern anti-arthritic agent.

## Materials and methods

### Preparation of RLE

Collection and authentication of RMF and LJF were the same as in a previous report^9^. Voucher specimens of the two herbs (RMF: YS6, LJF: JYH) were deposited at our School. As originally recorded in Tang dynasty of China, patients with inflammatory diseases were asked to drink rice wine in which the two ingredient herbs of RL had been soaked^10^. This suggests that active constituents in the formula are lipophilic. For use in this work, we extracted RL with different concentrations of ethanol and compared the effects of the extracts on nitric oxide (NO) production in lipopolysaccharide (LPS)-stimulated macrophages. Results showed that among the extracts, RLE exhibited the most potent inhibitory effects (Fig. S1). RLE was extracted with absolute ethanol^20^; its yield was 15.2%. Contents of chlorogenic acid and gallic acid in RLE were quantified using an HPLC method (Fig. S2).

### TUNEL assays

Knee joints of CIA and normal rats that had been dosed with drugs or vehicle in a previous study^20^ were collected. All care and handling of animals were performed in accordance with the recommendations of the Committee on the Use of Human & Animal Subjects of the Hong Kong Baptist University. The protocol was approved by the Department of Health, Hong Kong [Reference number: (19-21) in DH/SHS/8/2/6 Pt. 3]. The ARRIVE guidelines for reporting animal research were fully implemented. In that study, there were three groups of RLE intragastrically (i.g.)-dosed rats; of those three groups, results from only two (330 mg/kg group and 660 mg/kg group) were used in this study. The two groups exhibited stronger anti-arthritic effects than the 165 mg/kg group^20^. We also included the normal control (0.5% carboxymethyl cellulose-Na, i.g.), model (0.5% carboxymethyl cellulose-Na, i.g.) and positive control (2.5 mg/kg of indomethacin, i.g.) groups in the present study. There were 8 rats in each group, for a total of 48 rats. The joints were fixed and sectioned as described previously^28^. The sections were processed for TUNEL assays (In Situ Apoptosis Detection Kit, USA) following the manufacturer's instructions. Cryosection imaging was performed as described^29^. TUNEL assay results were quantified by counting the DAPI and TUNEL-positive cells in three individual fields using ImageJ software. Percentages of TUNEL-positive cells in synovia of rat joints were calculated using the following equation: TUNEL-positive cells (%) = TUNEL-positive cells/DAPI-positive cells × 100%. The researcher (J-Y Wu) assigned to calculate percentages of TUNEL-positive cells was blinded to the experimental design.

## IHC staining

Knee joints of rats were fixed and sectioned. IHC staining was performed as described^29^. The primary cleaved caspase-3 antibody was bought from Cell Signaling Technology (#9579; USA) and the secondary antibody was bought from Abcam (#ab205719; Cambridge, UK). Cryosection imaging was performed as described in the TUNEL assay section. IHC staining results were quantified by counting the DAPI and cleaved caspase-3-positive cells in three individual fields using ImageJ software. Percentages of cleaved caspase-3-positive cells in synovia of rat joints were calculated using the following equation: cleaved caspase-3-positive cells (%) = cleaved caspase-3-positive cells/DAPI-positive cells × 100%. The researcher (J-Y Wu) assigned to calculate percentages of cleaved caspase-3-positive cells was blinded to the experimental design.

### Cell culture

Patient-derived synoviocytes RA-FLS were obtained from Cell Applications (USA). L929 and MIHA cells were purchased from American Type Culture Collection (USA). Cells were maintained in high glucose Dulbecco’s modified Eagle’s medium (GIBCO, MA, USA) supplemented with 10% fetal bovine serum (GIBCO) and 1% penicillin/streptomycin (GIBCO) at 37 °C in a humidified atmosphere of 5% CO_2_.

### Cell Counting Kit-8 (CCK-8) and crystal violet staining assays

CCK-8 assay^30^ was performed to detect RLE’s effects on the proliferation of RA-FLS, L929 cells and MIHA cells. 100 ng/mL each of IL-6/sIL-6R (PeproTech, NY, USA) was used to stimulate cells in this study. RA-FLS seeded in 96-well plates (5,000 cells/well) were pre-treated with RLE (0–600 μg/mL) or 100 μM of indomethacin (positive control) for 1 h and then treated with IL-6/sIL-6R for 24 or 48 h. L929 or MIHA cells seeded in 96-well plates (5,000 cells/well) were pre-treated with RLE (0–600 μg/mL) for 1 h and then treated with IL-6/sIL-6R for 24 h. Crystal violet staining assay^31^ was also performed to determine RLE’s effects on the proliferation of RA-FLS. Cells seeded in 60-mm dishes (2 × 10^5^ cells/dish) were pre-treated with RLE (0, 150, 300 μg/mL) for 1 h and then treated with IL-6/sIL-6R for 48 h.

### Apoptosis assay

RLE’s apoptotic effects on RA-FLS were measured by Annexin V-FITC/PI double staining^32^ using an Apoptosis Detection Kit (#ab14085; Abcam). Synoviocytes seeded in 6-well plates (1 × 10^5^ cells/well) were pre-treated with RLE (0, 150, 300 μg/mL) for 1 h and then treated with IL-6/sIL-6R for 24 h. Flow cytometric analysis was performed using a BD Accuri C6 flow cytometer (BD Biosciences, USA).

### Western blot analysis

Proteins were prepared from ankle synovial tissues of rats and cultured cells. Whole cell, cytoplasmic and nuclear extracts were prepared and immunoblotting was conducted as described previously^32^. Primary antibodies JAK2 (#3230), STAT3 (#12640), cleaved caspase 9 (#52873), phospho-JAK2 (Tyr1007/1008, #3771), phospho-STAT3 (Tyr705, #9131), Myeloid cell leukemia 1 (Mcl-1, #94296) and lamin B1 (#12586) were obtained from Cell Signaling Technology; GAPDH (#sc365062)and B-cell lymphoma 2 (Bcl-2, #sc7382) antibodies were obtained from Santa Cruz Biotechnology (USA). Cleaved caspase 3 (#ab2302) and HRP-conjugated secondary antibodies (#ab7090, #ab97040) were obtained from Abcam.

### Cell transient transduction and viability assays of transduced cells

Adenovirus expressing GFP-Flag-tagged STAT3C (Ad-STAT3C) and control adenovirus (Ad-Empty vector) with green fluorescent protein (GFP) as a transduction indicator were prepared by Vigene Biosciences (Shandong, China). STAT3C (A662C, N664C mutant) is a constitutively active STAT3 variant^23^. RA-FLS were seeded in 100-mm dishes (6 × 10^5^ cells/dish) and transduced with Ad-STAT3C (7.8 × 10^7^ pfu/mL) or Ad-Empty vector (7.8 × 10^7^ pfu/mL); after 24 h, the supernatant was discarded and replaced with fresh medium. After a 12-h incubation, transduced RA-FLS were used for cell viability assays. RA-FLS^Empty vector^ and RA-FLS^STAT3C^ seeded in 96-well plates (5,000 cells/well) were separately pre-treated with RLE (0, 150, 300 μg/mL) for 1 h and then treated with IL-6/sIL-6R for 48 h. Cell viability was assessed using CCK-8 assays^30^. Viability of RLE-untreated RA-FLS^Empty vector^ or RLE-untreated RA-FLS^STAT3C^ was regarded as 100%.

### Statistical analysis

GraphPad software (GraphPad Prism 5.0, USA) was used for statistical analyses. Data are presented as mean ± standard deviation (SD), and were analyzed using one-way ANOVA followed by Dunnett's test. *P* < 0.05 was considered statistically significant.

## Supplementary Information

Supplementary Information
