# Supplementary material for: A two-herb formula inhibits hyperproliferation of rheumatoid arthritis fibroblast-like synoviocytes
Source: Sci Rep. 2021 Feb 16;11:3850. doi: 10.1038/s41598-021-83435-2 (PMC7886911; doi:10.1038/s41598-021-83435-2)
Supplement: Supplementary file 1 — Supplementary Information [file 41598_2021_83435_MOESM1_ESM.pdf]

A two-herb formula inhibits hyperproliferation of rheumatoid arthritis fibroblast-like synoviocytes

-Supplementary Figures-

Ying-Jie Chen[1,2,†], Yu-Xi Liu [1,2,†], Jia-Ying Wu [1,2,†], Chun-Yu Li[2], Min-Min Tang[2], Lu Bai[2], Xiu-Qiong Fu[1,2], Jun-Kui Li[1,2], Ji-Yao Chou[1,2], Cheng-Le Yin[1,2], Ya-Ping Wang[1,2], Jing-Xuan Bai[1,2], Ying Wu[1,2], Xiao-Qi Wang[1,2] and Zhi-Ling Yu[1,2,3,*]

[1]Research and Development Centre for Natural Health Products, HKBU Shenzhen Research Institute and Continuing Education, Shenzhen, China. [2]Centre for Cancer and Inflammation Research, School of Chinese Medicine, Hong Kong Baptist University, Kowloon Tong, Hong Kong, China. [3]Consun Chinese Medicines Research Centre for Renal Diseases, School of Chinese Medicine, Hong Kong Baptist University, Kowloon Tong, Hong Kong, China. [†]These authors contributed equally: Y.-J.C., Y.-X.L. and J.-Y.W. [*]Correspondence and requests for materials should be addressed to Z.-L.Y. (email: zlyu@hkbu.edu.hk)

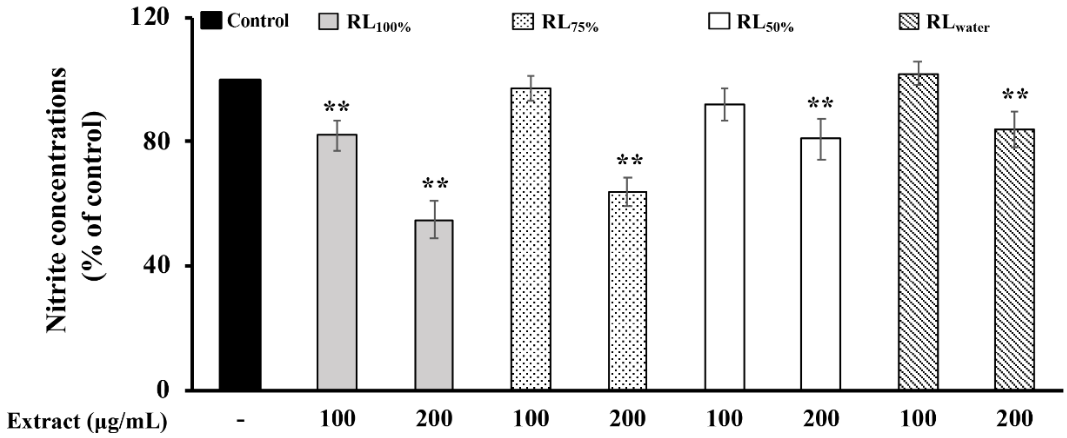

Figure S1: Effects of RL extracts on nitric oxide (NO) production in lipopolysaccharide (LPS)-stimulated RAW264.7 cells. RAW264.7 cells were pre-treated with or without RL extracts for 1 h and then incubated in the presence of LPS (1 μg/mL; *Escherichia coli* 0111:B4, Sigma, St. Louis, MO, USA) for 24 h. Nitrite levels in culture supernatant were determined by Griess assays. The value of the control group (LPS mono treatment group) was set at 100%. All extracts prepared with water and different concentrations of ethanol showed a concentration-dependent inhibition on NO production (reflected by the level of nitrite). Among the four extracts, $RL_{100\%}$ (in the manuscript, the extract was named as RLE) exhibited the most potent suppression on NO production. Values are the means ± SD (n = 3). ** P <0.01 compared with LPS mono treatment.

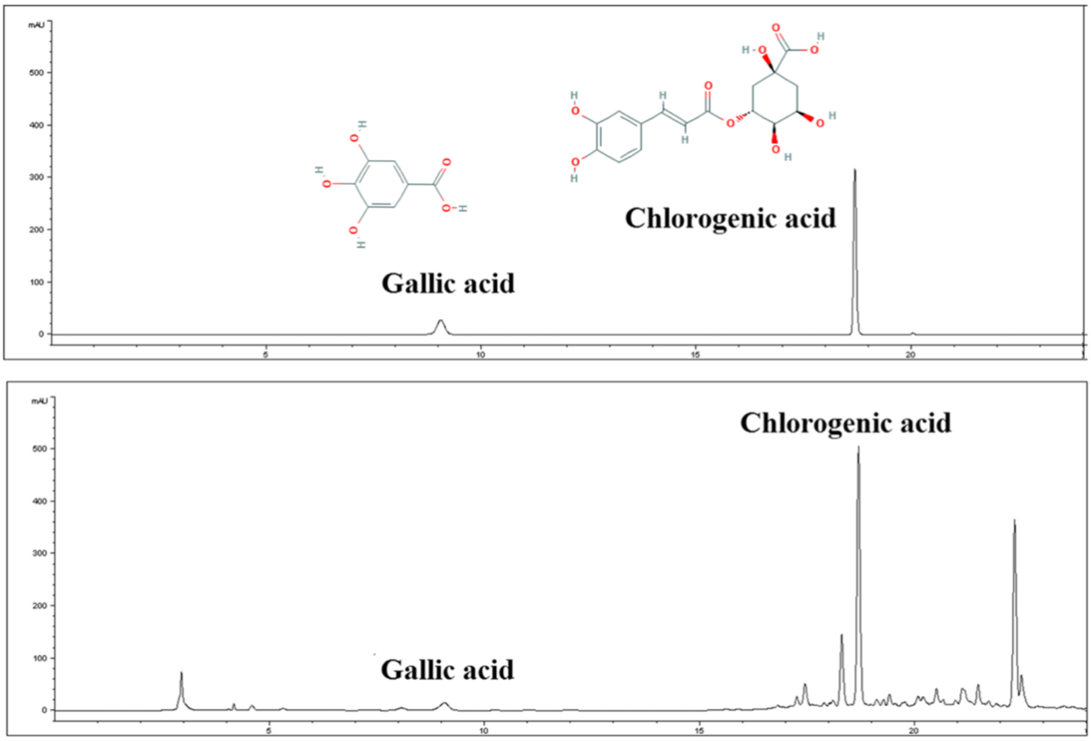

Figure S2: An HPLC method developed for quality control of RLE. HPLC chromatograms of chemical markers gallic acid and chlorogenic acid (upper panel) and RLE (lower panel). HPLC analysis was performed on an Agilent 1200 system equipped with Diode-array detector and coupled with a C_{18} column (4.6×250 mm^2, 5 µm; Agilent Technologies). Elution was performed with a mobile phase of A (0.2% (v/v) phosphoric acid) and B (acetonitrile) under a gradient program of 95% A at 0-9.5 min, 62% A at 9.5-20 min, followed by 60% A at 20-30 min. The flow rate was 1.0 mL/min. The column temperature was set at 25 °C. Sample injection volume was 10 µl in each test. Since both gallic acid and chlorogenic acid have a prominent absorption around 272 nm in the UV spectrum, 272 nm was chosen as the reference wavelength. The HPLC chromatogram showed that gallic acid and chlorogenic acid were present in RLE. The mean content of gallic acid was 763 µg per gram of RLE, and that of chlorogenic acid was 26,399 µg per gram of RLE. This is the same as in a previous report (*J Ethnopharmacol*. **153**: 922-927).

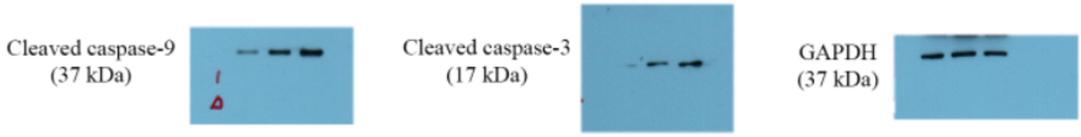

Figure S3: Full-length blots of Figure 3B. Cleaved caspase-3 and GAPDH were cropped from different parts of one gel, and cleaved caspase-9 was cropped from another gel. The bands were showed on different films because of different exposure time.

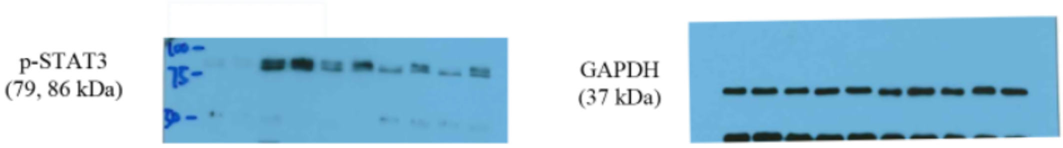

Figure S4: Full-length blots of Figure 4. STAT3 and GAPDH were cropped from different parts of a gel. The bands were showed on different films because of different exposure time.

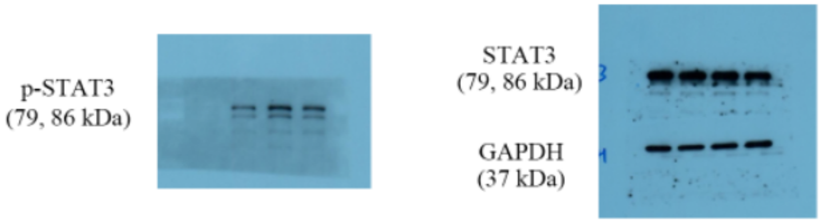

Figure S5: Full-length blots of Figure 5A. STAT3 and GAPDH were cropped from different parts of one gel, and p-STAT3 was cropped from another gel.The bands were showed on different films because of different exposure time.

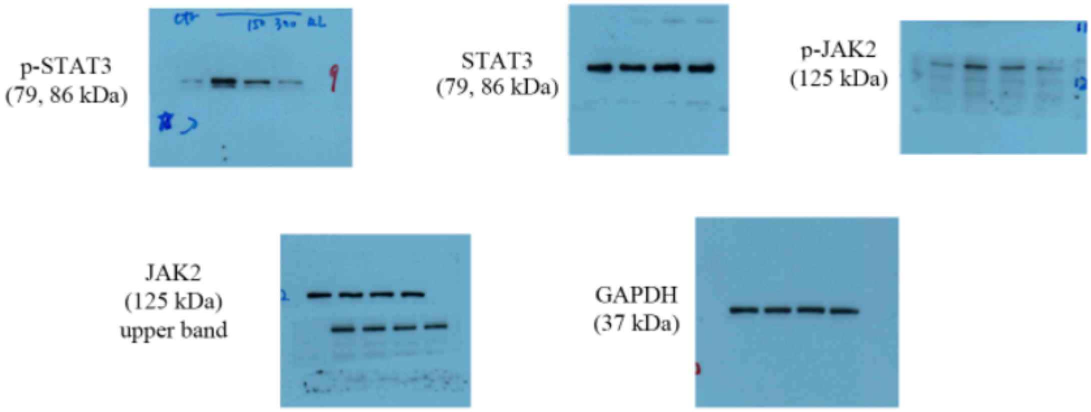

Figure S6: Full-length blots of Figure 5B. STAT3, JAK2 and GAPDH were cropped from different parts of one gel, and p-STAT3 and p-JAK2 were cropped from different parts of another gel. The bands were showed on different films because of different exposure time.

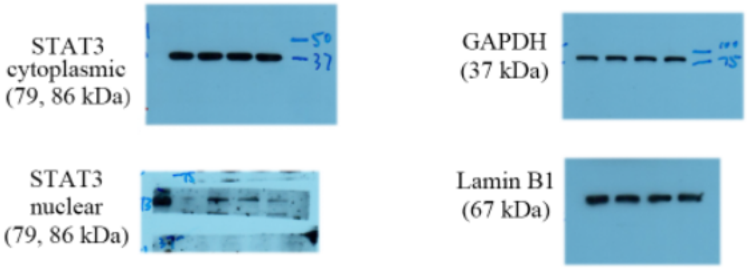

Figure S7: Full-length blots of Figure 5C. Nuclear STAT3 and Lamin B1 were respectively cropped from different gels, and cytoplasmic STAT3 and GAPDH were cropped from different parts of another gel. The bands were showed on different films because of different exposure time.

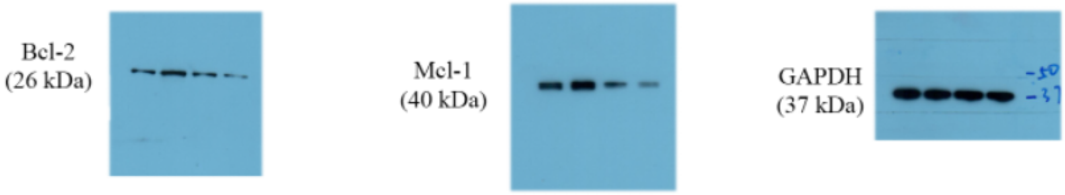

Figure S8: Full-length blots of Figure 5D. Bcl-2 and Mcl-1 were cropped from different parts of one gel, and GAPDH was cropped from another gel. The bands were showed on different films because of different exposure time.

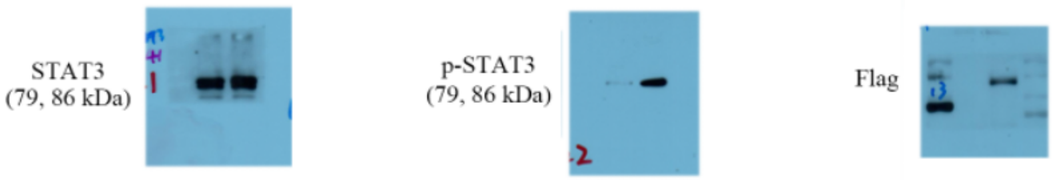

Figure S9: Full-length blots of Figure 6A. STAT3, p-STAT3 and Flag were respectively cropped from different gels. The bands were showed on different films because of different exposure time.
